# Unraveling Gene Fusions for Drug Repositioning in High-Risk Neuroblastoma

**DOI:** 10.3389/fphar.2021.608778

**Published:** 2021-04-23

**Authors:** Zhichao Liu, Xi Chen, Ruth Roberts, Ruili Huang, Mike Mikailov, Weida Tong

**Affiliations:** ^1^ Division of Bioinformatics and Biostatistics, National Center for Toxicological Research, US Food and Drug Administration, Jefferson, AR, United States; ^2^ ApconiX, BioHub at Alderley Park, Alderley Edge, United Kingdom; ^3^ University of Birmingham, Edgbaston, Birmingham, United Kingdom; ^4^ National Center for Advancing Translational Sciences, National Institutes of Health, Rockville, MD, United States; ^5^ Office of Science and Engineering Labs, Center for Devices and Radiological Health, US Food and Drug Administration, Silver Spring, MD, United States

**Keywords:** neuroblastoma, structural variants, gene fusions, next-generation sequencing, drug repositioning, precision medicine 3

## Abstract

High-risk neuroblastoma (NB) remains a significant therapeutic challenge facing current pediatric oncology patients. Structural variants such as gene fusions have shown an initial promise in enhancing mechanistic understanding of NB and improving survival rates. In this study, we performed a comprehensive *in silico* investigation on the translational ability of gene fusions for patient stratification and treatment development for high-risk NB patients. Specifically, three state-of-the-art gene fusion detection algorithms, including ChimeraScan, SOAPfuse, and TopHat-Fusion, were employed to identify the fusion transcripts in a RNA-seq data set of 498 neuroblastoma patients. Then, the 176 high-risk patients were further stratified into four different subgroups based on gene fusion profiles. Furthermore, Kaplan-Meier survival analysis was performed, and differentially expressed genes (DEGs) for the redefined high-risk group were extracted and functionally analyzed. Finally, repositioning candidates were enriched in each patient subgroup with drug transcriptomic profiles from the LINCS L1000 Connectivity Map. We found the number of identified gene fusions was increased from clinical the low-risk stage to the high-risk stage. Although the technical concordance of fusion detection algorithms was suboptimal, they have a similar biological relevance concerning perturbed pathways and regulated DEGs. The gene fusion profiles could be utilized to redefine high-risk patient subgroups with significant onset age of NB, which yielded the improved survival curves (Log-rank *p* value ≤ 0.05). Out of 48 enriched repositioning candidates, 45 (93.8%) have antitumor potency, and 24 (50%) were confirmed with either on-going clinical trials or literature reports. The gene fusion profiles have a discrimination power for redefining patient subgroups in high-risk NB and facilitate precision medicine-based drug repositioning implementation.

## Introduction

Neuroblastoma (NB) is the most common and deadly pediatric malignancy, and the average age of patients is about 1–2°years at diagnosis (Matthay et al., 2016; Fletcher et al., 2018). Approximately 70% of NB patients have a metastatic disease with a less than 30% event-free survival rate (Moroz et al., 2011). Several therapy and treatment options, such as immunotherapeutic strategies, local irradiation, autologous stem cell transplantation (ASCT) combined with chemotherapy, have improved the survival rate of NB patients. However, a substantial number of NB patients, particularly in the high-risk group, still suffer profound treatment-related morbidity (Semeraro et al., 2015). Therefore, advanced treatment options are still urgently needed to improve the survival rate while eliminating adverse events.

The molecular understanding of high-risk NB is mostly elusive. It becomes significant hurdles to advance NB prognosis and therapy development (Almstedt et al., 2020). Beyond the frequently detected gene alternations such as *ALK* activations (Mossé et al., 2008), *MYCN* amplification (Zeid et al., 2018), and LMO1 expression (Wang et al., 2011), advancement in sequencing technologies provided a more in-depth and width view of the molecular basis of NB (Pugh et al., 2013; Bueno et al., 2016; Boeva et al., 2017; Ma et al., 2018). More and more complex genetic events such as gene fusions have been identified and could differential high-risk NB patients (Rorie et al., 2004; Mertens et al., 2015; Peifer et al., 2015). For example, (Peifer et al., 2015) discovered recurrent genomic rearrangements of the telomerase reverse transcriptase gene (*TERT*), occurring only in high-risk NB patients. Furthermore, *TERT*-related fusions could be used to define a new patient subgroup in high-risk NB with adverse clinical outcomes. These promising findings trigger deeper thinking on how to translate these genetic findings into therapy development.

Gene fusion is a result of structural variants (SVs), including insertion, deletion, inversion, and translocation that joins the two separate transcripts. Commonality and diversity of gene fusions in cancer were discussed elsewhere (Lee et al., 2017; Xu et al., 2018; Gao et al., 2018; Hu et al., 2018; Picco et al., 2019). A lot of computational tools have been developed to detect fusion transcripts based on DNA/RNA sequencing (Kim and Salzberg, 2011; Jia et al., 2013; Beccuti et al., 2014; Davidson et al., 2015; Heyer et al., 2019). Some comparative studies have also been conducted to prioritize the fusion detection tools based on statistical measures such as precision and F-scores (Wang et al., 2013; Liu et al., 2016; Kumar et al., 2016; Zhang et al., 2016). The conclusion drawn from these comparative studies mainly suggested combined top performance callers generate consensus results for further experimental verification (Liu et al., 2016; Kumar et al., 2016). Fusion detection algorithms with different mathematical equations and hypothesis behind, it is still an open question on how to anchor and apply these algorithms based on biological relevance. Furthermore, two tumors rarely shared the same gene fusions due to tumor heterogeneity, which limited the discrimination power for stratification of cancer into informative subtypes based on the individual fused transcript. The question has been raised on whether the patient gene fusion profiles could be used to regroup the patients, which are predictive of clinical outcomes such as patient survival, therapy response, and tumor pathology.

Moreover, oncogenic gene fusion not only expands our understanding of tumor biology but provides possible therapeutic targets for treatment development. For example, Imatinib and ponatinib were approved US FDA to treat chronic myeloid leukemia (CML), which targets BCR-ABL1 fusion (Druker, 2008). Moreover, crizotinib/ceritinib inhibiting *ALK* fusion was approved to treat non–small cell lung cancer (Mertens et al., 2015). However, these approved drugs are mainly tyrosine kinase inhibitor (TKI) inhibitors, which suffers some severe adverse drug reactions (ADRs) such as cardiotoxicity and drug-induced liver injury (Liu et al., 2017; Xu et al., 2018). It is interesting to explore the probability of utilizing transcriptomic response, interplayed with gene fusions for a distinct patient subgroup for alternative treatment development while minimizing toxicity.

**FIGURE 1:**
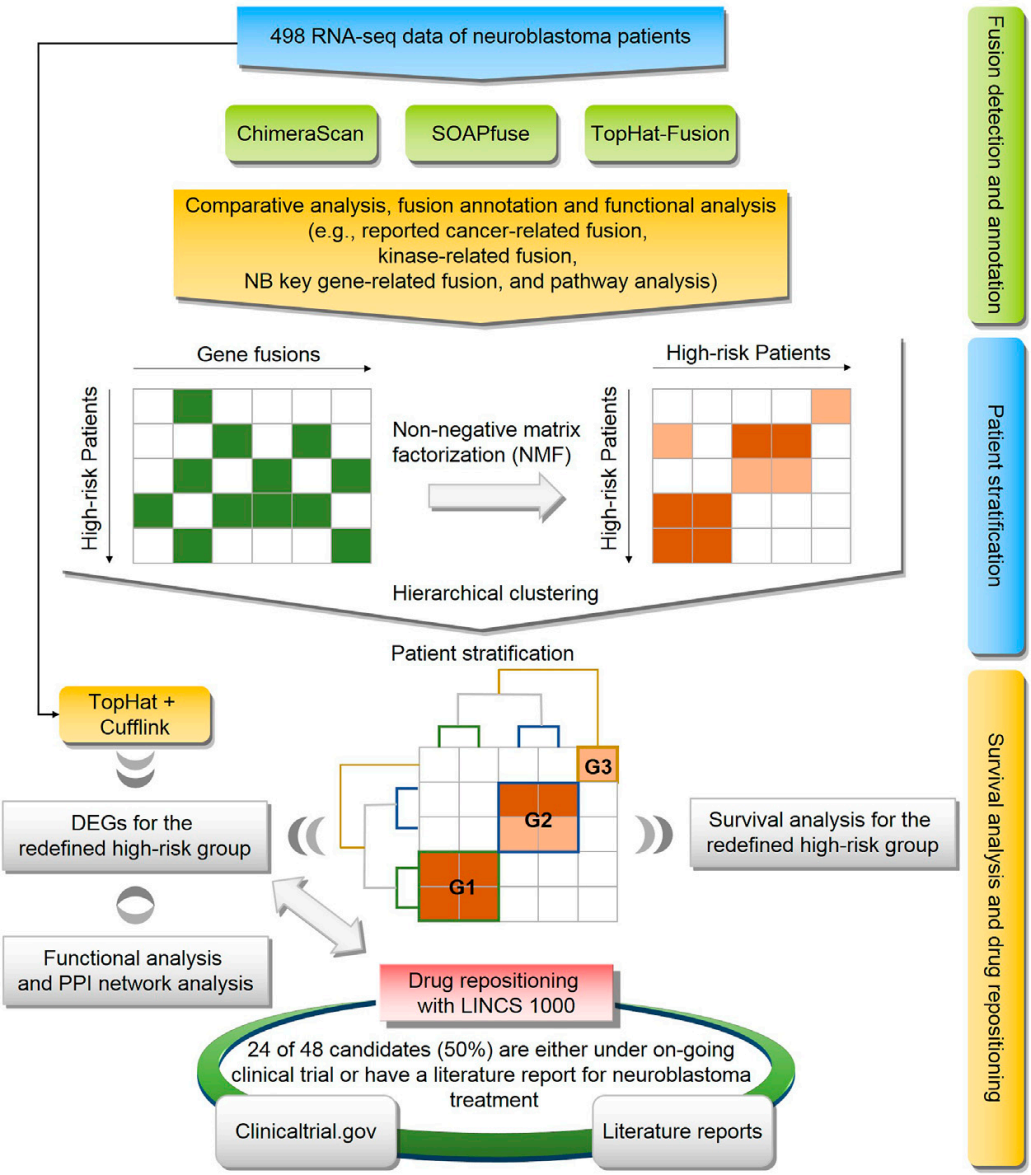
Flowchart of study: the workflow consists of three components including **(1)** fusion detection by three algorithms (i.e., ChimeraScan, SOAPfuse, and TopHat-Fusion) and fusion annotation by publicly available database and pathway analysis; **(2)** The high-risk patients were regrouped based on gene fusion profiles using non-negative matrix factorization (NMF) and hierarchical cluster analysis (HCA); **(3)** Survival analysis and drug repositioning for the redefined patient subgroup.

To explore the potential solutions for these unsolved questions, we conducted a comprehensive genomic analysis of 498 human NB cases (Figure 1). First, A landscape of gene fusions was presented by using three state-of-art fusion calling algorithms, including ChimeraScan (Iyer et al., 2011), SOAPfuse (Jia et al., 2013), and TopHat-Fusion (Kim and Salzberg, 2011). Then, high-risk NB patients were regrouped based on detected gene fusion profiles and evaluated by survival analysis. Next, differentially expressed genes (DEGs) of the redefined high-risk NB patient subgroups were extracted and functionally analyzed. Finally, Repositioning candidates for the redefined high-risk subgroups were enriched with a large-scale of transcriptomic profiles in LINCS L1000 Connectivity Map (CMap). The proposed framework provides a promising approach to translate novel genetic findings into therapy development.

## Materials and Methods

### RNA-Seq Dataset of Neuroblastoma

The tumor samples of 498NB patients were enrolled from seven countries under the consent of respective clinical trials. The range of patients at diagnosis was from 0 to 295.5°months. The patients were classified based on the International Neuroblastoma Staging System (INSS, https://www.cancer.org/cancer/neuroblastoma/detection-diagnosis-staging/staging.html) and the MYCN-amplified (MNA) was measured. The ratio of MNA patients and total patients in each stage: stage 1 (3/121), stage 2 (5/78), stage 3 (15/63), stage 4 (65/183), and stage 4 (4/53). Furthermore, 176 patients were classified as high-risk ones based on the Revised International Neuroblastoma Response Criteria. The clinical characteristics of 498 NB patients were listed in Supplementary Table S1.

The detailed sample preparation was described elsewhere (Oberthuer et al., 2006). Briefly, the patients’ tumor samples were manually checked by a pathologist and ensure the sample contains at least 60% of tumor content. The total RNA was then isolated from 30 to 60 mg of snap-frozen tissue obtained before cytotoxic treatment using the FastPrep FP120 cell disruptor (Qbiogene-Inc, Carlsbad, CA) and the TRIzol reagent (Invitrogen, Karlsruhe, Germany). Last, the RNA integrity was assessed, and the samples were selected with an RNA integrity number of more than 7.5. The RNA-seq data of 498 primary NB samples were generated in FDA Sequencing Quality Control (SEQC) phase I project. The raw RNA-seq data could be downloaded from the Gene Expression Omnibus (GEO, https://www.ncbi.nlm.nih.gov/geo/) database with series accession number GSE62564 (Zhang et al., 2015). The purified mRNA was extracted from total RNA using Dynabeads^®^ mRNA Purification Kit (Invitrogen), and ERCC RNA spike-in was injected based on the user guide for RNA sequencing. Then, the non-stranded TruSeqs™ protocol was used to conduct Library preparation. Next, clusters were generated based on the TruSeq PE Cluster Kit v3 reagent preparation guide. Last. High-throughput shotgun sequencing was performed on the Illumina HiSeq 2000 platform with the paired-end 100-bp reads. A total of 30,753,066,000 reads were produced, which enabled high coverage of the entire genome spectrum of NB. The average reads per sample was 6.1790e+07 ± 9.5474e+06, and the reads were distributed in a range of 39270986 to 10442490 (see Supplementary Figure S1). More detailed information on sequencing data generation was described elsewhere (Zhang et al., 2015).

### Detection of Transcript Expression and Fusion Transcripts

There are more than 20 state-of-art fusion transcript detection tools (Liu et al., 2016). In this study, we applied the three most cited tools, including TopHat-Fusion (Kim and Salzberg, 2011), ChimeraScan (Iyer et al., 2011), and SOAPfuse (Jia et al., 2013), to detect fusion transcripts from the RNA-seq data of 498 primary NB samples. In this study, we used the human reference genome sequence (hg19, downloaded from the UCSC Genome Browser: http://hgdownload.soe.ucsc.edu/downloads.html#human) to detect the transcript-level expression and fusion transcripts.

To generate a transcript-level expression, we used TopHat2 v2.1.0 (Kim et al., 2013) to align the raw reads to the UCSC human genome and quantified the expression levels of all the transcripts with FPKM (Fragments Per Kilobase of transcript per Million fragments mapped) values using Cufflinks v2.2.1 (Trapnell et al., 2010). All the parameters in the pipelines of alignment and quantification were set as default. The expressions at transcription-level were further summarized to the annotated genes in the RefSeq database (https://www.ncbi.nlm.nih.gov/refseq/) by using the highest FPKM value in those of transcripts as the expression of a gene when there were multiple transcripts corresponded to one gene (Beccuti et al., 2014).

There are many parameters in different fusion transcript detection algorithms, which have a significant impact on calling performance. However, optimization of parameter setting for each algorithm with different datasets is far beyond the scope of our paper. Therefore, we mainly followed the default parameter setting in each algorithm.

### TopHat-Fusion

For TopHat-Fusion, all raw reads in FASTQ files were first aligned to the reference genome by using the TopHat2 v2.1.0 (Kim et al., 2013). Then, the initially unmapped reads were split into small segments and remapped to the reference genome for identifying the initial fusion candidates by TopHat-Fusion. Lastly, the fusions, for which the number of fusion spanning reads was higher than five and the sum of the fusion spanning reads, and the supporting mate pairs were greater than 10, were kept as candidate fusion transcripts.

### ChimeraScan

The ChimeraScan pipeline utilized Bowtie (version 1.1.2) to align the raw reads to the reference genome (Langmead and Salzberg, 2012). The subsequent procedures of nominating the candidate fusions, detecting the spanning reads, and filtering the false positives were conducted with the default settings.

### SOAPfuse

For SOAPfuse (Li et al., 2009), the raw reads were mapped to the reference genome by using the SOAP2 (version 2.21) algorithm. The single-end and paired-end mapped reads were kept for identifying the candidate gene fusions. The unmapped reads were then aligned to the annotated transcripts (Ensemble release), and the mapped reads were retained. Finally, the unmapped reads in the second step were iteratively trimmed and realigned to the annotated transcripts until the length of the reads was less than 30 nucleotides. The reads still unmapped to the annotated transcripts were filtered out. All the aligned reads in the three steps were used to detect the gene fusions by seeking the span-reads. The maximum hits for each span-read, and the filter parameters for identifying the gene fusions, were set as default.

### Gene Fusion Annotation

The detected fusion transcripts were annotated by the following strategies. First, the reported fusion transcripts were curated and combined based on three public resources, including the Catalogue of Somatic Mutations in *Cancer* (COSMIC, https://cancer.sanger.ac.uk/cosmic/download) (Forbes et al., 2008), Tumor Fusion Gene Data Portal (http://tumorfusions.org/) (Xu et al., 2018), and ChimerDB 3.0 (https://academic.oup.com/nar/article/45/D1/D784/2605708) (Lee et al., 2017). Second, a list of genes associated with neuroblastoma risk was curated by literature survey by querying against PubMed and other databases using key words “genes” and “neuroblastoma”. Third, a file of human protein kinases was downloaded from UniProt (https://www.uniprot.org/docs/pkinfam). All the annotation data sets were listed in Supplementary Table S2.

### Fusion Transcripts-Based Stratification and Survival Analysis

The 176 high-risk NB patients were stratified into new subgroups based on the detected fusion transcript profiles. First, the patient-fusion transcript profiles matrix was constructed based on fusion transcripts detected from each algorithm. Then, NMF was used to decompose the patient-fusion transcript profiles matrix (***F***: 176 patients × unique number of fusion transcripts) into two matrices. 1) patient subgroup assignment (***W***: 176 patients × *k* subgroups) 2) fusion transcript assignment (***H***: k subgroups × unique number of fusion transcripts). This procedure was repeated 500 times. Consequently, the patient-patient relationship matrix (176 patients × 176 patients) was generated, and each cell of the matrix represent the probability of any two patients assigned to the same patient subgroup. Subsequently, the Hierarchical clustering analysis was used to create the consensus assignment of patients into *k* subgroups. Finally, Kaplan-Meier survival analysis was conducted for the comparisons between the subgroups, and the *p* values were calculated using the Log-rank test. All the procedures were performed in R (version 3.4.1) with the packages *NMF v0.21.0*, *ggplot2 v2.2.1*, *survminer v0.4.2*, and *survival v2.4.3*.

### Importance Fusions for Distigusing the Patients Subgroups

To further investigate the important fusions and classification performance of gene fusions for redefined patient groups, we employed the XGboost binary classifier (Chen and Guestrin, 2016). XGBoost (Extreme Gradient Boosting) is an ensemble machine learning algorithm for regression and classification problems based on the Gradient Boosting Decision Tree (GBDT), which has been widely applied in biomedical applications (Ji et al., 2019). Specifically, the 176 high-risk patients were stratified into patient subgroups, and we developed the XGboost classifier on the gene fusion profiles and their redefined patient groups. The important gene fusion profiles and performance metrics (i.e., The area under the receiving operating characteristic curve (AUC)) based on 100-run 5-fold cross-validation were calculated. The calculation was performed in R (version 3.4.1) with packages xgboost version 1.3.2. The detailed hyperparameters, including binary: logistic objective, max-depth 6, step size of each boosting step 50, were used.

### Differentially Expressed Genes in Patient Subgroups

To identify the differentially expressed genes (DEGs), we separately compared the transcript profiles of the patients in *k* subgroups with those of the patients in the control group, in which the survival days of the patients were longer than the median survival days among the stages 1 and 2. The DEGs were finally identified using the R packages *limma* and *edgeR* with an adjusted *p* value less than 0.05 as a cut-off value (Robinson and Smyth, 2008). The genes in the DEG list were ranked by their fold changes in descending order and the top/down 500 genes were extracted for further analysis. The functional analysis of extracted DEGs in each patient subgroup was conducted by using The Database for Annotation, Visualization, and Integrated Discovery (DAVID, https://david.ncifcrf.gov/) [44].

### Enrichment of Repositioning Candidates

Drug-induced transcriptional profiles of NIH LINCS project (http://www.lincsproject.org/) (Corsello et al., 2017) were employed to enrich repositioning candidates specifically for each patient subgroup. The hypothesis behind genome-based repositioning is that if the drug signature is reversely correlated with the disease signature, the drug could be potentially used to treat the diseases. For LINCS data, LINCS L1000 characteristic direction signatures search engine (L1000CDS^2^) was used to reversely compare the DEG in each patient subgroup to the drug transcriptional signatures in LINCS project (Duan et al., 2016). The L1000CDS^2^ used data sets including LINCS L1000 level 3 normalized data and level 5 moderated Z-scores (MODZ), which were downloaded from lincscloud.org and GEO (GSE70138). There are a total of transcriptomic profiles generated from 98 cell lines.

### Immune Cell Gene Signatures

Although neuroblastoma is typically considered to be an immunologically ‘cold’ tumor (Szanto et al., 2020), several studies have demonstrated the presence of tumor-infiltrating lymphocytes (e.g., T cells and NK cells), in human neuroblastoma tumors (Coughlin et al., 2006; Hishiki et al., 2018; Wienke et al., 2021). Therefore, we further investigated the immune-related gene expression in the refined patient subgroup. Immune cell gene expression data in mouse cell lines and tissues were extracted from the Immunological Genome Project (ImmGen) (Heng and Painter, 2008). The pre-processing and normalization of data were described previously (Painter et al., 2011; Kidd et al., 2015). Specifically, 304 differential state gene expression (e.g., fold change values) covering 11,153 mapped ortholog human Entrez gene ids were generated between two steady-state profiles from 221 unique immunological cell types (stored at https://github.com/iguana128/Gene-fusion_NB). In this study, we ranked order gene expression profile from high to low based on fold change values for each of 304 immune-related states. Then, the top/down 500 genes in each immunological state were selected as DEGs for further analysis.

### Code Availability

The scripts of RNA-seq analysis, data analysis, and data curation were listed in the GitHub (https://github.com/iguana128/Gene-fusion_NB). Furthermore, the scripts for generating all the figures and tables were provided as well.

## Results

### Overview of Gene Fusion Transcripts

**FIGURE 2:**
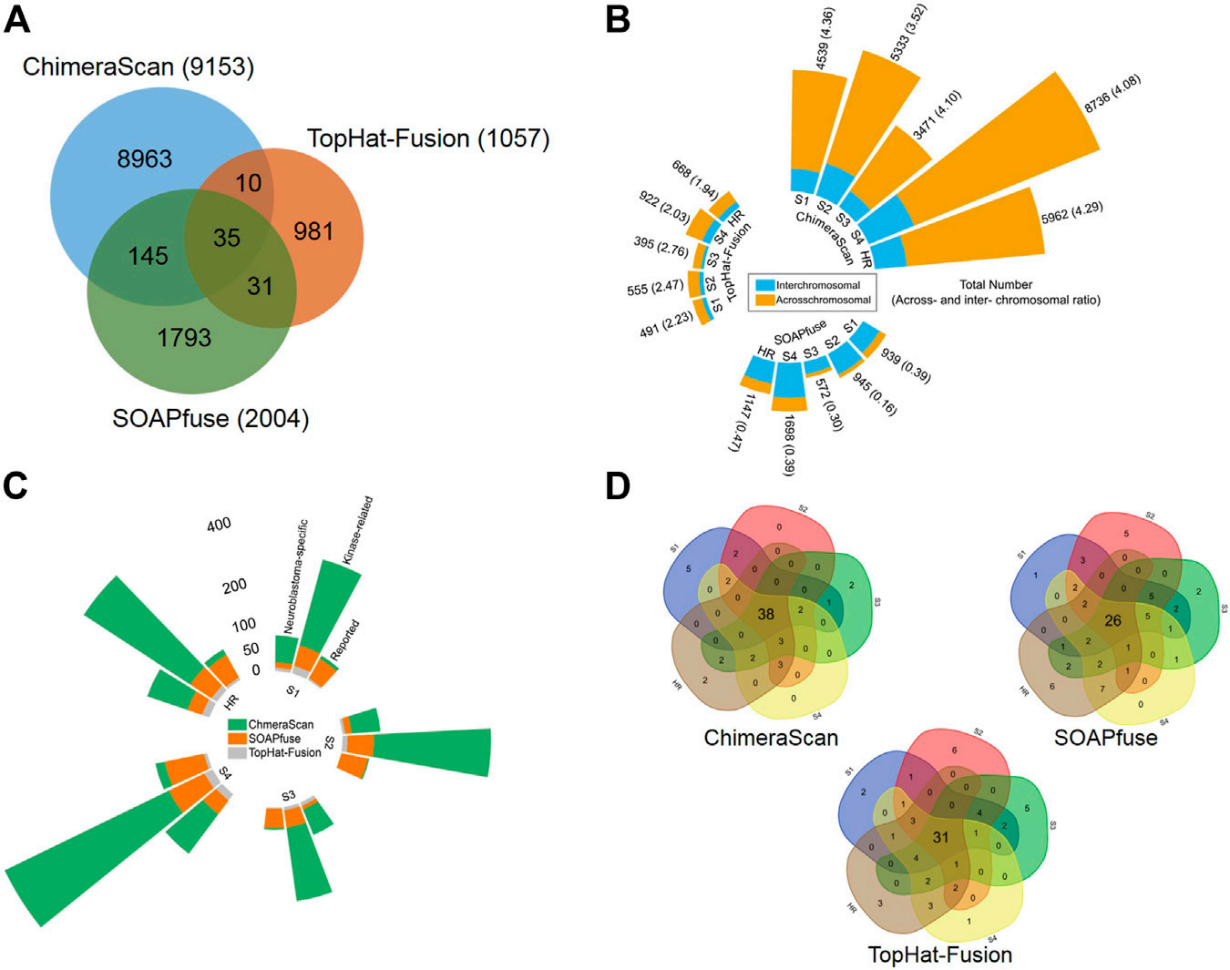
Comparative analysis and annotation of identified gene fusions by three algorithms, including ChimeraScan, SOAPfuse, and TopHat-Fusion: **(A)** a Venn diagram of detected fusions among three algorithms; **(B)** Distribution of detected gene fusions in different INSS clinical stages and high-risk group. The yellow and blue color represents the inter- and across-chromosomal gene fusion, respectively; **(C)** The detected fusions in each clinical stage were further annotated by reported cancer-related gene fusions, kinase-related fusions, and neuroblastoma key gene-related gene fusions; **(D)** The high-frequency gene fusions for each algorithm were extracted across the different clinical stages.

Three algorithms, including ChimeraScan (Iyer et al., 2011), SOAPfuse (Jia et al., 2013), and TopHat-Fusion (Kim and Salzberg, 2011), were employed to harbor gene fusion transcripts among 498 human NB cases. A total of 9,153 (i.e., 9,153/498 = 18.37 per patient), 2,004 (i.e., 2,004/498 = 4.02 per patient), and 1,057 (i.e., 1,057/498 = 2.12 per patient) unique gene fusions were detected by ChimeraScan, SOAPfuse, and TopHat-Fusion, respectively (Supplementary Table S3). We found 47.07% (4,308/9,153), 59.28% (1,188/2,004), and 43.61% (461/1,057) of total detected fusions only specific to the individual patient for ChimeraScan, SOAPfuse, and TopHat-Fusion, respectively. ChimeraScan is more sensitive to detect more gene fusions than another two algorithms. There were only 221 gene fusion transcripts identified by at least two algorithms, which represents the divergence of fusion detection algorithms (Figure 2A). We further investigated the distribution of detected gene fusions across the patient subgroups defined by using the International Neuroblastoma Staging System (INSS). Specifically, the number of identified gene fusions were increased from the early stages (stage 1 ∼ stage 3) to late stages (stage 4 and high-risk group), indicating the higher stage has more complex tumor compositions. For ChimeraScan and TopHat-Fusion, more across-chromosomal fusions were detected than that of inter-chromosomal fusions. However, SOAPfuse harbored more inter-chromosomal fusion transcripts (Figure 2B). Furthermore, the concordances among the three algorithms for individual patients also tended to be increased in patients in stage 4 and high-risk groups, although overall concordances were suboptimal (less than 5% overlapped ratio) (Supplementary Figure S2).

The detected gene fusions from the three algorithms were further annotated by the curated knowledge concerning reported cancer-related genes fusions, kinase protein family, and neuroblastoma related key genes. Similarly, patients from late stages (stage 4 and high-risk group) enriched more regarding reported cancer-related fusions, kinase-related fusions, and neuroblastoma key genes related fusions. The total number of annotated fusions was increased from TopHat-Fusion to SOAPfuse and ChimeraScan (Figure 2C). Table 1 summarized the annotated fusions identified by the three algorithms. SOAPfuse detected more reported cancer-related fusions (90) than that of ChimeraScan (26) and TopHat-Fusion (10). *Axon guidance* pathway was enriched by the genes involving reported cancer-related gene fusions from both ChimeraScan and SOAPfuse. However, the enriched pathways (e.g., *Hippo signaling pathway* and *Oxytocin signaling pathway*) based on reported gene fusions identified by TopHat-Fusion were distinct. *MAPK signaling pathway* was enriched by kinase-related fusions from both ChimeraScan and TopHat-Fusion, although the involved gene fusions were entirely different. It indicated that fusion detection algorithms have a relatively higher similarity in the biological levels. It was interesting that some neuroblastoma related essential genes are more susceptible to form gene fusions. For example, ChimeraScan was more sensitive to *ALK* related fusions, while SOAPfuse and TopHat-Fusion detected more *MYCN* and *LOM1* related fusions. *DDX1* associated fusions were identified by all three algorithms.

**TABLE 1:** Annotated gene fusions in different fusion detection algorithms.

Fusion detection algorithms	Number of identified fusions	Representative fusions	Involved KEGG pathways	Involved genes	*p* values
Reported cancer-related gene fusions					
ChimeraScan	26	TRA@_TRA@; CNRIP1_PPP3R1; CAMTA2_SPAG7; C11orf48_INTS5 BMPR1B_PDLIM5; DDX17_DMC1; GCC2_RANBP2; GPR128_TFG; POR_RHBDD2; SIPA1L3_WDR62	hsa04360: Axon guidance	NCK1, MET, PPP3R1, PPP3CC	5.5E-3
			hsa04660: T cell receptor signaling pathway	NCK1, PPP3R1, PPP3CC	3.5E-2
SOAPfuse	90	CLSTN1_CTNNBIP1; CYB5R4_RIPPLY2; ACTN4_EIF3K; ANKDD1A_PLEKHO2; BAG2_ZNF451; EIF4E3_FOXP1; CNRIP1_PPP3R1; TBCEL_TECTA; ADIPOR1_CYB5R1; POR_RHBDD2	hsa04520: Adherens junction	ACTN4, MET, LMO7, CTNND1, IQGAP1	7.2E-3
			hsa04360: Axon guidance	ABLIM2, NCK1, MET, PPP3R1, PPP3CC	4.9E-2
TopHat-fusion	10	DDX1_NBAS; PLB1_PPP1CB; ALK_GALNT14; CCND1_ORAOV1; CNRIP1_PPP3R1; DNAJB4_FUBP1; EIF4E3_FOXP1; BAIAP2_TBCD; BMPR1B_PDLIM5; KDM4A_ST3GAL3	hsa04390: Hippo signaling pathway	CCND1, BMPR1B, PPP1CB	1.2E-2
			hsa04921: Oxytocin signaling pathway	CCND1, BMPR1B, PPP1CB	1.3E-2
Kinase-related gene fusions					
ChimeraScan	422	AK095450_FER; FLJ25037_KSR1; MAST2_TIMM23; PHKG2_TH; DAPK3_MYO9A; MXD4_PKN1; AF070581_PAK3; MAP3K15_SNORD10; STK38L_TRIM8; AK124179_PASK	hsa04010: MAPK signaling pathway	FGFR2, FGFR1, FGFR3, MAPKAPK5, MAP4K2, MAPKAPK3, MKNK2, MKNK1, AKT1, MAP3K6, MAP3K3, PAK1, AKT3, MAP2K5, AKT2, PRKCA, TAOK2, MAP2K2, NLK, MAP2K3, MAP2K4, TAOK3, NR4A1, PRKCG, MAPK11, MAPK10, STK4, FLNA, STK3, PRKCB, MAPK1, MAP4K4, MAPK12, MAPK13, NTRK1, MAPK14, PDGFRB, MAPK9, MAPK7, MAP3K12	2.7E-18
			hsa04012: ErbB signaling pathway	Prkca, ERBB3, MAP2K2, ERBB2, CAMK2G, MAP2K4, RPS6KB2, PRKCG, MAPK10, PRKCB, AKT1, MAPK1, PTK2, PAK3, GSK3B, MAPK9, PIK3CA, CAMK2B, PAK1, ABL1, CAMK2A, AKT3, AKT2	2.5E-15
SOAPfuse	93	LIMK2_RNF185; CHCHD2_PHKG1; PRKAA1_TTC33; FES_MAN2A2; BCKDK_KAT8; INSL3_JAK3; TAOK2_TMEM219; NTRK1_PEAR1; MAP2K5_SKOR1; ACVR2B_CNOT6	hsa04722: Neurotrophin signaling pathway	IRAK4, MAPK1, PDPK1, MAP2K1, RPS6KA2, CAMK2G, NTRK1, CAMK2B, MAPK7, AKT3, AKT2, MAP2K5	4.5E-8
			hsa04921: Oxytocin signaling pathway	MAPK1, MAP2K1, ROCK2, CAMK2G, PRKAA1, CAMK2B, EEF2, GNAS, MAPK7, SRC, PRKCB, CAMK1D, MAP2K5	8.7E-8
TopHat-fusion	29	IRAK3_RBMS1; STK24_STK24P1; LOC407835_MAP2K2; DAPK1_RPS29; BMX_HNRNPDL; C14orf166_MERTK; MAPK11_MAPK12; ENSG00000226049_TLK2; CDK4_TMEM132C; ALK_GALNT14	hsa04010: MAPK signaling pathway	PAK2, MAPK12, TAOK1, RPS6KA2, MAP2K2, MAP2K4, MAPK11	1.8E-4
			hsa04660: T cell receptor signaling pathway	PAK2, MAPK12, MAP2K2, MAPK11, CDK4	3.9E-4
Neuroblastoma key genes-related fusions					
ChimeraScan	138	CHD5_FOXI3; ALK_ANKS1A; ALK_USP11; CNOT3_DLK1; DLK1_FLJ00420; DLK1_KIAA0691; BC035411_DDX1; DDX1_SMA4; AX748330_RASSF7; PDGFA_SLC29A4	hsa04010: MAPK signaling pathway	PTPN7, BDNF, CACNG8, PDGFA, CACNG7, NTRK1, MAP2K4, PPP3R1, TP53, NR4A1, STK4, STK3	2.9E-5
			hsa04210: Apoptosis	TNFRSF10C, TNFRSF10B, NTRK1, PIK3CD, CASP8, TP53	3.1E-4
SOAPfuse	43	HOXC4_HOXC6; ENSG00000198353_HOXC6; LMO1_RIC3; NTRK1_PEAR1; KIF1B_PGD; DDX1_NBAS; CAMTA1_VAMP3; DDX1_MYCNOS; DDX1_MYCNUN; NPRL2_ZMYND10	hsa01130: Biosynthesis of antibiotics	ODC1, NME2, NME1-NME2, NME1, PGD	5.6E-3
			hsa00240: Pyrimidine metabolism	NME2, NME1-NME2, NME1	5.0E-2
TopHat-fusion	22	ANGPT2_MCPH1-AS1; FMO4_TOP1; EDARADD_ENO1; NME2_NME2P1; DDX1_NBAS; ALK_GALNT14; MYCN_NBAS; DDX1_MYCNUT; FOXR1_PAFAH1B2; HACE1_SCML4	hsa01130: Biosynthesis of antibiotics	ODC1, NME2, NME1-NME2, ENO1	5.1E-3
			hsa01100: Metabolic pathways	ODC1, NME2, NME1-NME2, PAFAH1B2, GALNT14, ENO1	4.6E-2

We further extracted the top fifty high-frequent gene fusions across the patients in each stage for the three algorithms (Figure 2D). There were 38, 26, and 31 high-frequency gene fusions that appeared in all clinical stages for ChimeraScan, SOAPfuse, and TopHat-Fusion, respectively (Supplementary Table S4). However, as mentioned above, substantial low-frequency gene fusions are more patient-specific.

### Fusion Transcripts-Based Stratification

**FIGURE 3:**
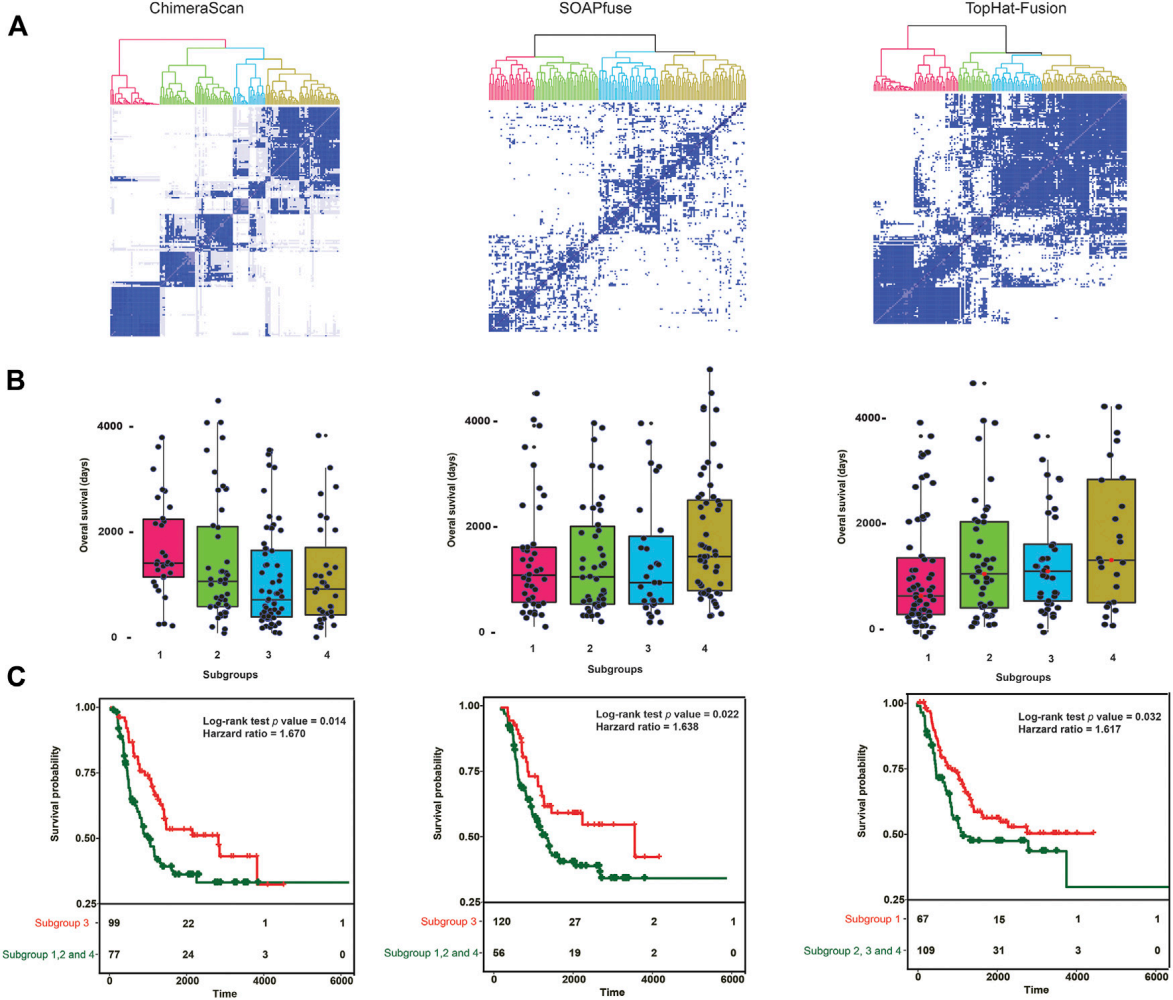
Gene fusion profile-based patient stratification and survival analysis: **(A)** hierarchical cluster analysis (HCA) for redefining patient subgroups in high-risk NB patient groups for each fusion detection algorithm. First, patient assignment matrices were generated from average results of 500 runs with nonnegative matrix factorization (NMF) with patient gene fusion profiles. Then, the HCA was performed on the patient assignment matrices to redefine the patient groups. The color denotes the redefined patient groups; **(B)** The overall survival time (Days) of patients in the redefined patient subgroups. The subgroup with lowest median survival time was considered as the redefined high-risk group; **(C)** Kaplan-Meier survival analysis was conducted between the redefined high-risk subgroups and the combination of other subgroups. Log-Rank *p* value and the hazard ratio was calculated.

To further investigate the discrimination power of gene fusion profiles, we stratified 176 high-risk NB patients into new subgroups based on the detected fusion transcripts in each of the three algorithms. Here, only fusions appeared more than one time in the high-risk group were employed. Consequently, the high-risk patient and gene fusion profiles matrices were constructed with dimensions 176 patients × 2,509 fusions, 176 patients × 446 fusions, and 176 patients × 351 fusions for ChimeraScan, SOAPfuse, and TopHat-Fusion, respectively. Then, 176 patients were divided into a predefined number of subtypes (*k* = 6, 6, and 3) by using average subtype assignment frequency from 500 runs of the NMF algorithm. The predefined subtype *k* was optimized by using cophenetic degree and sparseness parameters in NMF (Supplementary Figure S3). Then, patient subtype assignment matrices (176 patients × 176 patients) for each fusion detection method were further clustered by using hierarchical clustering analysis (HCA) (Figure 3A). The 176 patients were clustered into four subgroups for each fusion algorithm. The overall survival distribution of each subtype was illustrated in Figure 3B. It is illustrated that there existed one subgroup (number of patients = 99, 120, and 67) with significantly smaller overall survival time (median overall survival = 798, 883, and 735°days) than another three groups (median overall survival = 1,242, 1,241, and 1,229°days). It was found 39 patients were overlapped by the subgroups based on fusion profiles from ChimeraScan (39/99 = 39.4%), SOAPfuse (39/120 = 32.5%), and TopHat-Fusion (39/67 = 58.2%), respectively (Supplementary Figure S4). The Kaplan-Meier survival analysis showed significantly different survival times between the redefined patient subgroups with *p* values (0.014, 0.022, and 0.032) and hazard ratios (1.670, 1.638, and 1.617) for the three algorithms (Figure 3C; Supplementary Table S5). It was indicated that the fusion transcript profiles could be used to further distinguish the patients from the high-risk group with improved survival rates.

Table 2 listed the clinical characteristics of redefined high-risk patients. MYCN-amplified patients are more enriched in the refined high-risk patient group (73.7, 72.5, and 59.7% for ChimeraScan, SOAPfuse, and TopHat-Fusion, respectively). In contrast, the lower percentage of MYCN-amplified patients was classified into other subgroups (i.e., 24.7, 8.9, and 47.7% for ChimeraScan, SOAPfuse, and TopHat-Fusion, respectively). It was indicated that the redefined patient group might be MYCN-amplified, especially for ChimeraScan and SOAPfuse. Furthermore, the age of diagnosis was more than 18°months for over 92% of refined high-risk patients and 96% of patients in other groups, highlighting the early diagnosis of high-risk NB patients is very challenging. Additionally, a higher percentage of males were assigned into the redefined high-risk patient group, e.g., 57.5 and 62.7% for ChimeraScan and TopHat-Fusion, respectively. However, a higher rate of females was classified into other subgroups. The detail clinical information for redeinfed high-risk patients were listed in Supplementary Table S5.

**TABLE 2:** Clinical characteristics of redefined high-risk patients

Clinical characteristics	Group	Algorithm	Number	Percentage of total
MYCN status				
Normal	Redefined high-risk	ChimeraScan	26	27.3%
		SOAPfuse	33	27.5%
		TopHat-fusion	27	40.3%
	Others	ChimeraScan	57	74.0%
		SOAPfuse	50	89.3%
		TopHat-fusion	56	51.3%
Amplified	Redefined high-risk	ChimeraScan	73	73.7%
		SOAPfuse	87	72.5%
		TopHat-fusion	40	59.7%
	Others	ChimeraScan	19	24.7%
		SOAPfuse	5	8.9%
		TopHat-fusion	52	47.7%
N.A.	Redefined high-risk	ChimeraScan	0	0
		SOAPfuse	0	0
		TopHat-fusion	0	0
	Others	ChimeraScan	1	1.3%
		SOAPfuse	1	1.8%
		TopHat-fusion	1	1.0%
Age at diagnosis				
<18°months	Redefined high-risk	ChimeraScan	6	6.1%
		SOAPfuse	9	7.5%
		TopHat-fusion	5	7.5%
	Others	ChimeraScan	3	3.9%
		SOAPfuse	0	0%
		TopHat-fusion	4	3.7%
>18°months	Redefined high-risk	ChimeraScan	93	93.9%
		SOAPfuse	111	92.5%
		TopHat-fusion	62	92.5%
	Others	ChimeraScan	74	96.1%
		SOAPfuse	56	100%
		TopHat-fusion	105	96.3%
Sex				
Male	Redefined high-risk	ChimeraScan	57	57.5%
		SOAPfuse	52	43.3%
		TopHat-fusion	42	62.7%
	Others	ChimeraScan	54	70.1%
		SOAPfuse	43	76.8%
		TopHat-fusion	69	63.3%
Female	Redefined high-risk	ChimeraScan	42	42.5%
		SOAPfuse	68	56.7%
		TopHat-fusion	25	37.7%
	Others	ChimeraScan	23	29.9%
		SOAPfuse	13	23.2%
		TopHat-fusion	40	26.7%

**FIGURE 4:**
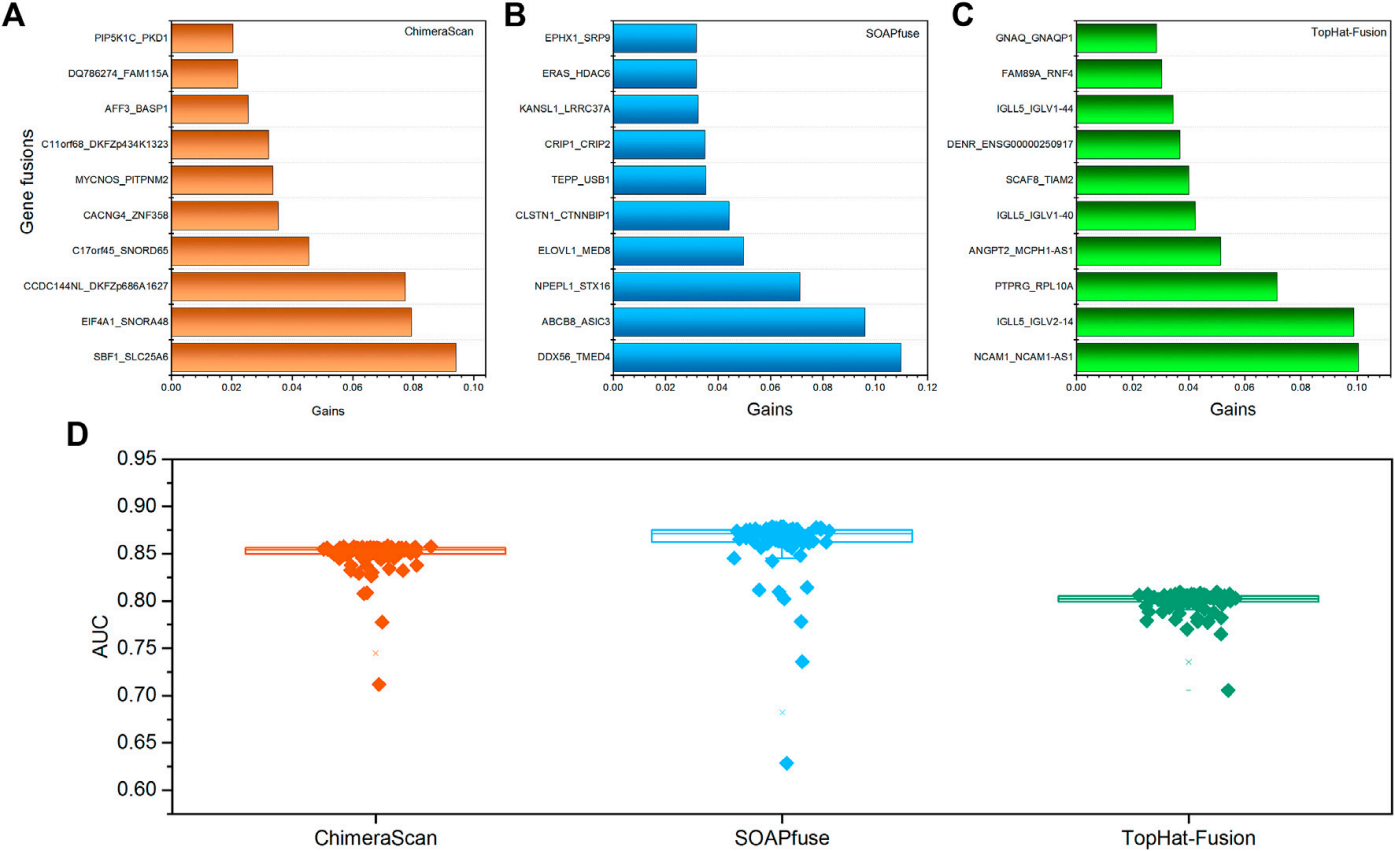
The top 10 important gene fusions and performance of the XGboost classifier for redefined high-risk patient subgroup: **(A–C)** the top 10 gene fusions extracted from XGboost model for ChimeraScan, SOAPfuse, and TopHat-Fusion, respectively; **(D)** the average AUC of 100-run 5-fold cross-validations (CVs) of the XGBoost model.

To further investigate the important gene fusion profiles that could distinguish the high-risk group patients, we developed an XGboost binary classifier. Figure 4 illustrated the top 10 important features derived from XGboost classifiers for the three fusion detection tools. Furthermore, the average AUC values of 100-run 5-fold cross-validations were ranked with the following order: SOAPfuse (0.862 ± 0.031) > ChimeraScan (0.849 ± 0.018) > TopHat-Fusion (0.799 ± 0.013). The high AUC and small stand deviation among 100 5-fold cross-validation results indicated the reliable classification results could be obtained based on the gene fusion profiles derived from the three detection tools.

### Differentially Expressed Genes Associated With Patient Subgroups

**FIGURE 5:**
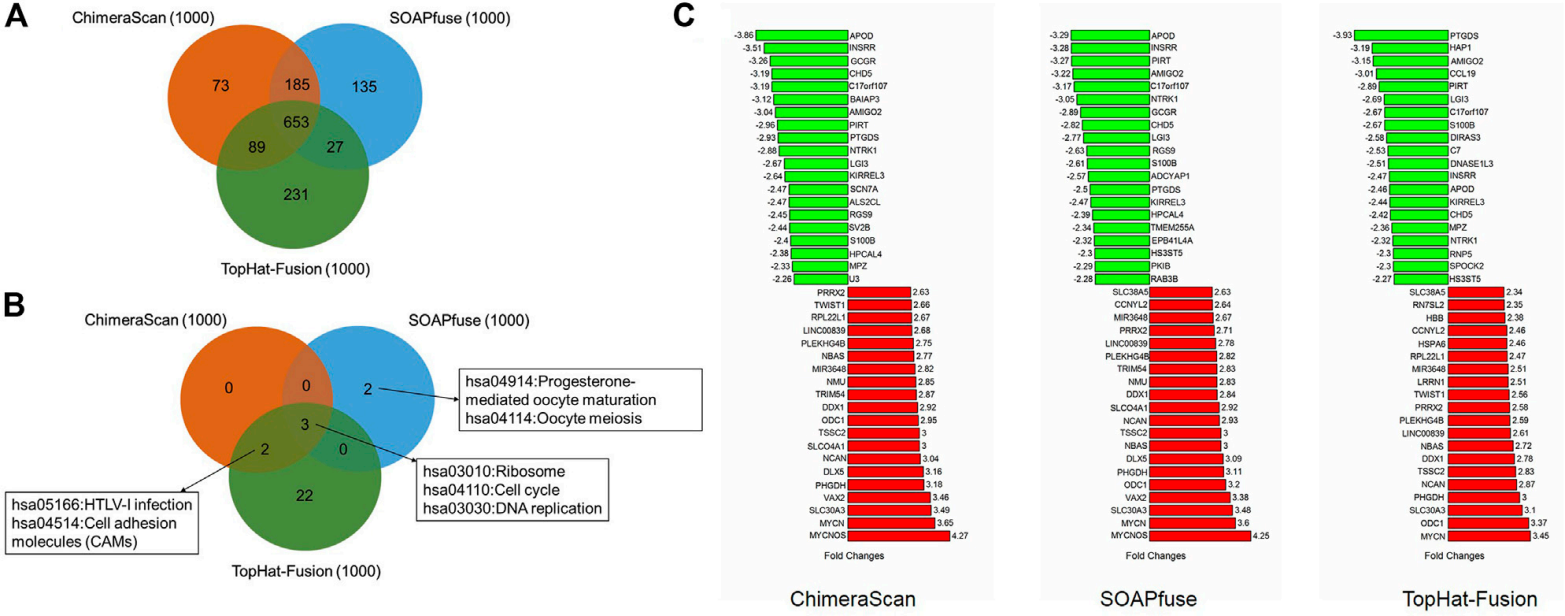
The comparative analysis of the differential expressed genes (DEGs) and related KEGG pathways regulated by the redefined high-risk group: **(A)** a Venn diagram of DEGs regulated by the redefined high-risk group for the three fusion detection algorithms; **(B)** a Venn diagram of KEGG pathways enriched by DEGs; **(C)** the top 20 up- and down-regulated genes of DEGs derived from the three algorithms.

We next sought for the DEGs associated with the redefined high-risk subgroups and examined their underlying mechanism. The DEGs were generated by comparing the transcript profiles between the patients in the redefined high-risk subgroup, and patients with the overall survival days were longer than the median survival days in stages 1 and 2. Here, the top 500 up-and down-regulated genes were extracted (Supplementary Table S6). The overlapped DEGs associated with refined high-risk subgroups from the three algorithms occupied 65.3% of 1,000 DEGs for each fusion detection method (Figure 5A). The shared KEGG pathways enriched by using DEGs were illustrated in Figure 5B. Three KEGG pathways, including *Ribosome*, *Cell cycle*, and *DNA replication,* were enriched by all the three algorithms. The DEG from TopHat-Fusion enriched more immune-related pathways such as *Primary immunodeficiency* and *an Intestinal immune network for IgA production* (Supplementary Table S7). Figure 5C highlighted the top ten up- and down-regulated genes associated with refined high-risk subgroups. *MYCN*, *MYCNOS*, and *SLC30A3* were the most up-regulated genes, while *APOD*, *INSRR*, and *PIRT* were the most down-regulated genes across the three algorithms. Those genes have been reported to play a different regulation role in NB development (Huang and Weiss, 2013; Olsson et al., 2016). Notably, *MYCN* has been found in ∼25% of high-risk NB patients and correlated with poor diagnosis (Huang and Weiss, 2013).

**FIGURE 6:**
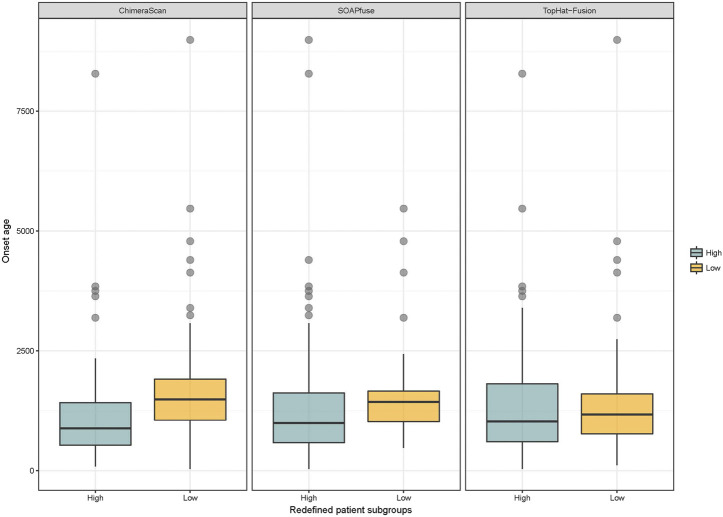
Distribution of onset age in the redefined high-risk and low-risk NB patients: blue and yellow colors represent the redefined high-risk and low-risk patient subgroups, respectively. The student’s t-test was used to generated *p* value.

**FIGURE 7:**
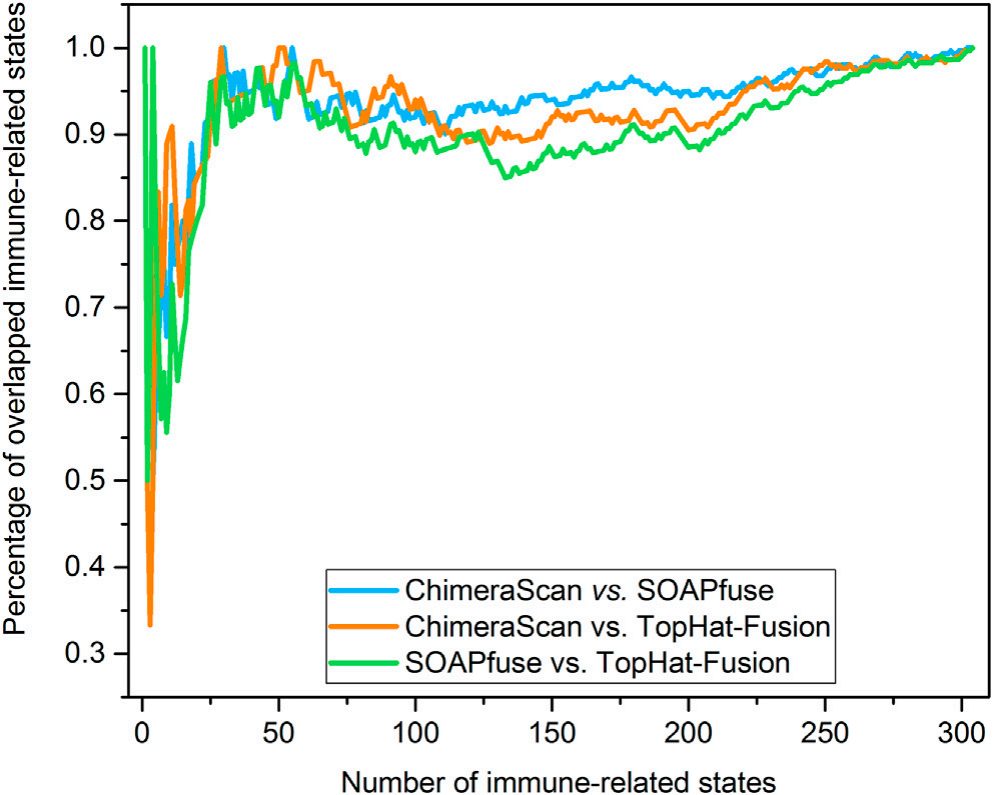
Percentage of overlapped immune-related cell types: the immune-related cell types were enriched and rank-ordered from high to low by comparing the DEG regulated by the redefined high-risk subgroup for each fusion detection algorithm and gene signatures of immune-related cell type from ImmGen (https://www.immgen.org/). Then, the percentage of overlapped enriched immune-related was calculated between any two rank-ordered immune-related cell type lists. The top-ten enriched immune-related cell types were illustrated in the sub-table for each fusion detection algorithm.

Another interesting finding here is the onset age of NB in the redefined high-risk NB patient group is significantly lower than another three redefined subgroups for ChimeraScan and SOAPfuse. It is indicated that the development of an adaptive immune system may play a role in neuroblastoma evolution (Figure 6). We further compared the DEG in a redefined high-risk group with the highly expressed gene signatures in 304 immune-related states obtained from the ImmGen (https://www.immgen.org/) (Heng and Painter, 2008). The similarities between DEG in the redefined high-risk subgroup and immune-related cell types were calculated and rank-ordered (Supplementary Table S8). The percentage of overlapped ranked immune-related cell types among the three fusion detection algorithms was illustrated in Figure 7. The enriched immune-related cell types were very similar among the three algorithms. Furthermore, we listed the top ten enriched immune-related cell types from each method and found T8 cell types dominated (Orentas et al., 2012).

### Repositioning Candidates for Redefined High-Risk Subgroup

We further explore the potential repositioning candidates that could be used to treat the patients in the redefined high-risk subgroup. The L1000 drug signatures were employed to compare the DEGs of the redefined high-risk group. LINCS L1000 characteristic direction signatures search engine (L1000CDS^2^) was applied to enrich Repositioning candidates. If the drug signatures were reversely correlated with DEG in a refined high-risk group, the drug was considered as potential repositioning candidates. Consequently, we got the top 50 repositioning candidates for patients in the redefined high-risk group from each fusion detection algorithms. Supplementary Table S9 listed the detailed information on repurposing candidates, including compound/time/dose/cell line enriched from L1000CDS^2^. Interestingly, a total of 37 Repositioning candidates were overlapped by at least two refined high-risk groups from different fusion detection algorithms (Supplementary Table S9).

To verify the enriched repositioning candidates, we conducted a two-step analysis. Table 3 compiles the candidates for the redefined high-risk groups defined by the three fusion detection algorithms. First, we queried clinical trial studies through http://clinicaltrial.gov (www.clinicaltrials.gov) to seek for the clinical evidence of repositioning candidates for the treatment of neuroblastoma. Notably, three repurposing candidates, including selumetinib, vemurafenib, and trametinib, were in clinical trials for potentially treating NB. Second, we further carried out a comprehensive literature survey through PubMed (https://www.ncbi.nlm.nih.gov/pubmed/) using the keywords (‘Repositioning candidate name’ AND ‘neuroblastoma’) to convey the finding here. Consequently, we further found 21 drugs have literature citations to support their potential use for NB patients. Collectively, we found 50% of candidates (24 (3 + 21) out of 48 repositioning candidates) to either have on-going clinical trial or literature supports for NB treatment. In total, 45 out of 48 repurposing candidates (93.8%) have been reported with antitumor potency.

**TABLE 3:** Summary information of Repositioning candidates for neuroblastoma patients in refined high-risk groups.

Repositioning candidates	Clinical phases	Mode of actions (MoAs)	Approved/Investigated therapeutic categories	Evidence	Confirmation sources
On-going clinical studies in clinicaltrial.gov					
selumetinib (BRD-K57080016)	Phase 2	Mitogen-activated protein kinase (MEK) inhibitor	Solid tumors such as neuroblastoma; non-Hodgkin lymphoma	Efficacy evaluation of selumetinib in treating patients with solid tumors such as recurrent neuroblastoma, non-Hodgkin lymphoma, or histiocytic disorders with MAPK pathway activation mutations that have spread to other places in the body and have come back or do not respond to treatment. (A pediatric MATCH treatment trial)	NCT03213691
vemurafenib (PLX4032, RG7204)	Phase 2	V600E mutated BRAF inhibitor	Melanomas; 8% of all solid tumors, including neuroblastoma, melanoma, colorectal, thyroid and other cancers	Efficacy evaluation of vemurafenib in treating patients with relapsed or refractory advanced solid tumors such as recurrent neuroblastoma, non-hodgkin lymphoma, or histiocytic disorders with BRAF V600 mutations (A pediatric MATCH treatment trial)	NCT03220035
trametinib	Phase 1	MEK inhibitor	Refractory solid tumors such as neuroblastoma; lymphomas; multiple myeloma	Next generation personalized neuroblastoma therapy by using trametinib	NCT02780128
Literature support from PubMed					
BRD-K68548958 (C646)	Investigational	Histone acetyltransferase p300 inhibitor	Prostate and lung cancers	Mouse *in vitro* (primary murine cortical neurons):C646 as a selective histone acetylation could regulate the expression of omega-3 polyunsaturated fatty acid, docosahexaenoic acid (DHA)-metabolizing enzyme Alox15 in neuroblastoma cells, which affect cognition and memory in brain development	Pmid: 29235036
BMS-536924	Investigational	Insulin-like growth factor-I receptor (IGF-IR)	Childhood sarcomas	Human *in vitro*: 28 sarcoma and neuroblastoma cell lines were screened for *in vitro* response to BMS-536924 to identify sensitive and resistant cell lines. Notably, Ewing's sarcoma, rhabdomyosarcoma, and neuroblastoma are more responsive to BMS-536924, suggesting these specific subtypes may represent potential targeted patient subpopulations for the IGF-IR inhibitor	Pmid: 19117999
mitoxantrone	Approved	DNA-reactive agent	Breast cancer; acute myeloid leukemia; non-Hodgkin’s lymphoma	Patient cohort study: A distinct side population (SP) was found in neuroblastoma cells from 15 of 23 patients (65%). These cells also expressed high levels of ABCG2 and ABCA3 transporter genes and had a greater capacity to expel cytotoxic drugs, such as mitoxantrone, resulting in better survival	PMID:15381773
MK-2206	Phase 2	Allosteric AKT inhibitor	Colorectal cancer; breast cancer; other solid tumors	Human *In vitro*: Combination of an allosteric akt inhibitor MK-2206 with etoposide or rapamycin enhances the antitumor growth effect in high-risk neuroblastoma patients	Pmid: 22550167
BRD-K65814004	Investigational	NADH/NADPH oxidase inhibitor	Antibiotics	Human *in vitro*: Neuroblastoma cell death was attenuated by ROS-scavengers and was dose-dependently inhibited by the NADPH oxidase inhibitor diphenyleneiodonium chloride (DPI)	Pmid: 16260066
BRD-A36630025 (SN-38)	Approved	a topoisomerase I inhibitor	Colon cancer, and small cell lung cancer	Mouse *in vivo*: Nanoparticle delivery of an SN-38 conjugate is more effective than irinotecan in a mouse model for treating neuroblastoma	Pmid: 25684664
DL-PDMP	Investigational	Glucosyltransferase inhibitor	Lewis lung carcinoma cell metastasis	Mouse *in vitro* (murine neuroblastoma cells): DL-PDMP is a potent inhibitor of glucosylceramide synthase, resulting in inhibition of the synthesis and shedding of gangliosides, which may contribute to the observed bone marrow depression in neuroblastoma patients	Pmid: 9809988
saracatinib (AZD-0530)	Phase 2	Dual kinase inhibitor, with selective actions as a src inhibitor and a bcr-abl tyrosine-kinase inhibitor	Alzheimer’s disease and schizophrenia	Human *in vitro* (neuroblastoma SKNSH cells): Iron depletion results in src kinase inhibition of saracatinib with associated cell cycle arrest in neuroblastoma cells	Pmid: 25825542
wortmannin	Phase 2	Covalent inhibitor of phosphoinositide 3-kinases (PI3Ks)	Recurrent glioblastoma	Human *in vitro* (neuroblastoma SKNSH cells): PI3K pathway inhibition down-regulates surviving expression and enhances TRAIL-mediated apoptosis in neuroblastomas. PI3K pathway may play a crucial role in neuroblastoma cell survival; therefore, treatment with inhibitors of PI3K such as LY294002 or wortmannin may provide potential novel therapeutic options	Pmid: 15065019
palbociclib	Approved	CDK4/6 inhibitor	ER-positive and HER2-negative breast cancer	Human *in vitro*: Selective inhibition of CDK4/6 using palbociclib may provide a new therapeutic option for treating neuroblastoma	Pmid: 26225123
naproxol (BRD-K34014345)	Approved	Nonsteroidal anti-inflammatory drugs (NSAIDs)	Inflammation	Human/rat *in vitro*: Protective effects of the former four non-steroidal anti-inflammatory drugs such as naproxol against apoptosis might be mainly due to their direct nitric oxide radical scavenging activities in neuronal cells	Pmid: 11259508
					Pmid: 15975708
Nutlin-3	Phase 1	*cis*-imidazoline analogs	Retinoblastoma	Human *in vitro*: Amplification or overexpression of MYCN sensitizes neuroblastoma cell lines with wild-type p53 to MDM2-p53 antagonists such as Nutlin-3 and MI-63, which may therefore be particularly effective in treating high-risk MYCN-amplified neuroblastoma	Pmid: 21725357
AS605240	Investigational	Selective PI3K inhibitors	Diabetics; rheumatoid arthritis; pulmonary fibrosis; cancer	Human/mouse *in vitro*: Phosphoinositide 3-kinases (PI3K) in selected neuroblastoma tumors with the inhibitor AS605240, which has been shown to express low toxicity and relative specificity for the PI3K species γ	Pmid: 20224967
gossypol (BRD-K19295594)	Investigational	Natural phenol derived from the cotton plant	Contraceptive and antimalarial	Human *in vitro*: Mcl1 appears as a predominant pro-survival protein contributing to chemoresistance in neuroblastoma, and Mcl1 inactivation may represent a novel therapeutic strategy. Optimization of compounds with higher Mcl1 affinity, or combination with additional Mcl1 antagonists such as gossypol, may enhance the clinical utility	Pmid: 19556859
ixazomib (MLN2238)	Approved	Proteasome inhibitor	Multiple myeloma	Mouse *in vivo*: ixazomib not only inhibits neuroblastoma cell proliferation and induces apoptosis but also enhances dox-induced cytotoxicity in neuroblastoma cells, suggesting that combination therapy including ixazomib with traditional therapeutic agents such as dox is a viable strategy that may achieve better outcomes for NB patients	Pmid: 27687684
canertinib (CI - 1033)	Discontinued	Irreversible tyrosine-kinase inhibitor	Various of cancer types	Human *in vitro*: Non-EGFR ERBB family members such as canertinib contribute directly to neuroblastoma growth and survival, and pan-ERBB inhibition represents a potential therapeutic target for treating neuroblastoma	Pmid: 20564646
AZD8055	Phase 1/2	mTOR inhibitor	Recurrent gliomas	Human *in vitro* and mouse *in vivo*: AZD8055 can induce cell cycle arrest, autophagy and apoptosis. AZD8055 strong antitumor activity on neuroblastoma *in vitro* and *in vivo*, which may be further investigated for treatment in clinical trials for high risk NB	Pmid: 29499203
teniposide	Approved	Podophyllotoxin derivatives	Acute lymphocytic leukemia (ALL)	Children with neuroblastoma have a significantly higher incidence of acute reactions to teniposide than patients with other malignancies (*p* = 0.008), and that these reactions cannot be prevented by premedication with antiallergic drugs	Pmid: 3857970
vorinostat	Approved	Histone deacetylases (HDAC) inhibitor	Cutaneous T cell lymphoma (CTCL)	Mouse *in vitro:* Vorinostat created a permissive tumor microenvironment (TME) for tumor-directed mAb therapy by increasing macrophage effector cells expressing high levels of fc-receptors (FcR) and decreasing the number and function of myeloid-derived suppressor cells (MDSC) in high-risk neuroblastoma	Pmid: 27471639
NVP-TAE684	Investigational	ALK inhibitor	Lung cancer and others	Human/mouse *in vitro*: The transforming potential of the putative gain-of-function ALK mutations as well as their signaling potential and the ability of two ATP-competitive inhibitors, crizotinib (PF-02341,066) and NVP-TAE684, to abrogate the activity of ALK for neuroblastoma patients	Pmid: 21838707
torin-2	Investigational	Selective mTOR inhibitor	Various of cancer types	Torin-2 with potency against both mTOR and PI3K was more effective in promoting cytotoxicity when combined with crizotinib. Our findings should enable a more precise selection of molecularly targeted agents for patients with ALK-mutated neuroblastoma	Pmid: 25228590

## Discussion

One of the critical aspects of precision medicine is to translate the novel genetic findings into therapy development. Structural variants (SVs) such as gene fusions have been identified in various tumor types, indicating some potential discriminative power to improve the patient survival curves. However, it was still elusive how to translate these novel genetic elements into therapy development. Several challenges become significant hurdles toward the practical application of novel genetic findings in a clinical setting. First, the inconsistency of gene fusions among the gene fusion detection algorithms still exists. Technically, several comprehensive comparison studies have been reported and suggested the consensus approach as a solution to improve reproducibility. However, the inconsistency among algorithms may be explained by biological relevance, which has not thoroughly investigated. Second, the discrimination power of gene fusion has only been assessed individually. The patient gene fusion profiles may provide a more robust solution to redefine the patient groups toward better survival rates. Finally, it is still a gap to utilize novel genetic findings into therapy development, although the possibility was always discussed elsewhere (Cully, 2015). We therefore aimed to explore the opportunity to apply patients’ gene fusion profiles to stratify the high-risk neuroblastoma patients into subgroups for improving the survival rate and implementing the precision medicine-based drug repositioning.

Overall, three popular gene fusion detection algorithms were comprehensively assessed with RNA-seq data of 498 NB patients. The technical reproducibility among the three algorithms was suboptimal, which reflected the interior difference of mathematical proof behind the algorithms. Moreover, the sample heterogenicity is still a significant factor for fusion detection algorithm selection. The number of gene fusions was correlated with clinical stages from low-risk to high-risk. The sensitivity of fusion detection algorithms was increased from TopHat-Fusion to SOAPfuse and ChimeraScan. Notably, the biological relevance of detected gene fusions from the three algorithms shared substantial similarity regarding regulating pathways and DEGs.

More importantly, the gene fusion profiles derived from each algorithm have a discrimination power to redefine patient subgroups in the high-risk group with improved survival rates with Log-Rank *p* values less than 0.05. Furthermore, highly overlapped repositioning candidates (37 out of 48 candidates) could be enriched based on DEGs from different algorithms, and 50% of repositioning candidates (24 out of 48 repositioning candidates) could be verified by on-going clinical studies and literature reports.

The detected gene fusions were annotated by the current knowledge (Table 1). For example, the *Axon guidance* pathway was enriched by genes involved in reported cancer-related gene fusions based on ChimeraScan and SOAPfuse. Axon guidance plays a central role in controlling neuronal migration and neuronal survival (Chédotal et al., 2005). The expression change of proteins such as slits, semaphorins, and netrins involved in the *Axon guidance* pathways induce the pathological changes in neural circuits which predisposed to neurological disorder in adult and NB in children (Van Battum et al., 2015). Another interesting finding here is some kinase-related fusions could perturb immune-related pathways such as the T cell receptor signaling pathway. It may help investigate the immune cell types regulated by gene fusion profiles in the redefined patient subgroups (Figures 5, 6). The abnormalities of immune systems in children with NB have been observed. However, the underlying mechanism is not fully understood. We found the onset age of NB patients in the redefined patient subgroup was significantly smaller than the others. Furthermore, some specific immune cell types could be enriched by all the three algorithms, which may provide more biological hints for better understanding the interplay between immune systems and pathogenesis of NB.

Several repurposing candidates hold a promise for further investigation on clinical usage for treating NB. Over 93% of enriched repositioning candidates were designed for anticancer purposes. For example, selumetinib and vemurafenib are in A Pediatric MATCH Treatment Trial (Phase 2) led by NIH to evaluate their efficacy on treating with solid tumors (e.g., NB), non-Hodgkin lymphoma, or histiocytic disorders with MAPK pathway activation mutations (clinicaltrial.gov IDs: NCT03213691 and NCT03220035). Trametinib, a MEK inhibitor, is in clinical phase 1 for its potential to treat refractory solid tumors, including NB (NCT02780128).

Some literature reports also show some preclinical evidence of repositioning candidates for NB treatments. One example is MK-2206, an allosteric AKT inhibitor is in clinical Phase 2 designed for colorectal cancer, breast cancer, and other solid tumors. Preclinical human *in vitro* studies suggested a combination of an allosteric Akt Inhibitor MK-2206 with etoposide or rapamycin to enhances the antitumor growth effect in high-risk NB patients (Li et al., 2012). Another example is AZD8055, an mTOR inhibitor, which was designed for treating recurrent gliomas in clinical phase 2. It was reported that AZD8055 could induce cell cycle arrest, autophagy, and apoptosis and had strong antitumor activity on NB in both human *in vitro* and mouse *in vivo* models. It may be worth further investigating for clinical application for high-risk NB treatment (Xu et al., 2018).

We also enriched one repositioning candidate named SN-38, which was initially approved for small cell lung cancer. SN-38 is a topoisomerase I inhibitor and the active metabolite of irinotecan. It has a solubility issue that makes it hard for patient administration. However, the efficacy was much better than anti-neuroblastoma drug irinotecan. A novel nano carrier-based strategy for tumor-targeted delivery of a prodrug of SN-38 was developed and verified in mouse xenografts, which solve the poor blood-brain barrier (BBB) concentration of SN-38 for neuroblastoma treatment (Iyer et al., 2015).

More than 20 different gene fusion detection tools based on RNA-seq data have been developed (Kumar et al., 2016; Haas et al., 2019). The performances of fusion detection tools on sensitivity, specificity, and required computational resource (e.g., memory size and computational time) varies among different datasets. It was suggested the critical influence of calling performance also highly relies on the RNA-Seq read length, read number, and the quality of the reads. The optimization of parameters in each pipeline based on the properties of their RNA-Seq datasets along statistical methods may improve the calling performance (Dehghannasiri et al., 2019). Another comparative study also suggested the consensus calling results from different detection tools may decrease the false-positive rates. The standardization of calling results from various detection tools is the key to avoiding the specific tools and establishing a consensus calling result (Liu et al., 2016). However, a ground truth set verified from orthogonal technologies was needed to assess the calling performances from different detection tools objectively. As a proof-of-concept paper, the points described above were out of the scope of the current study. However, we highly recommend further assessing the reliable fusion detection results from multiple detection tools to establish the reproducible and high-confidence fusion calling to enable a real-world application. It is worthwhile to consider some additional work to further enhance and confirm the findings in our studies. First, the current studies were focused on RNA-seq data. As our known, DNA-seq has been well-established for SVs detection. Further investigation on DNA-seq or combined with RNA-seq data could improve the precision of gene fusion detection and establish satisfactory technical reproducibility (Liu et al., 2019). The DNA-seq provides the unbiased characterization and most comprehensive of the potential gene fusions and tumor suppressor genes disrupted by genomic rearrangement. However, it requires in-depth coverage, ample storage, and long computational analysis time. The RNA-seq only sequences the genome regions that are transcribed and spliced into mature mRNA. Therefore, only relatively high expressed fusions can be detected. However, RNA-seq data requires less storage, space, and analysis time. Furthermore, the read length for RNA-seq data can be either short or long with different sequencing platforms (Schröder et al., 2019). Second, as a proof-of-concept study, we explored the discrimination power of gene fusion profiles and their potential for patient stratification and treatment development. However, an integrative approach by incorporating various genetic elements beyond mRNA could yield a better survival curve and more precise treatment development. Third, to reuse the drugs for pediatric tumor treatment is a challenging task. In this study, the enriched repositioning candidates are mainly anticancer candidates, which could serve as a good starting point. The further PK/PB property optimization, dosage adjustment, and safety profile prioritization should be taken into consideration for further investigation (Liu Z et al., 2016; Liu et al., 2017). Fourth, the unbalance number of collected neuroblastoma patient samples across different clinical predefined INSS cancer stages might influence the performance of gene-fusion-based patient stratification. The more consortium efforts with more balanced patients distribution in different cancer stages may further verify and improve the performance. Finally, in the current study, we only focused on three popular fusion detection algorithms to elaborate the points and establish the framework for translating genetic findings into therapy development. Other fusion detection algorithms and patient stratification strategies may also be considered to improve the results. In conclusion, we carried out an exploratory study to investigate how to apply genetic findings such as gene fusions for clinical applications. The framework developed it straightforward and may also serve as a strategy for treatment development for other diseases.

## Data Availability

The original contributions presented in the study are included in the article/Supplementary Material, further inquiries can be directed to the corresponding authors.
