# Supplementary figures and images for: Unraveling Gene Fusions for Drug Repositioning in High-Risk Neuroblastoma
Source: Front Pharmacol. 2021 Apr 23;12:608778. doi: 10.3389/fphar.2021.608778 (PMC8105087; doi:10.3389/fphar.2021.608778)

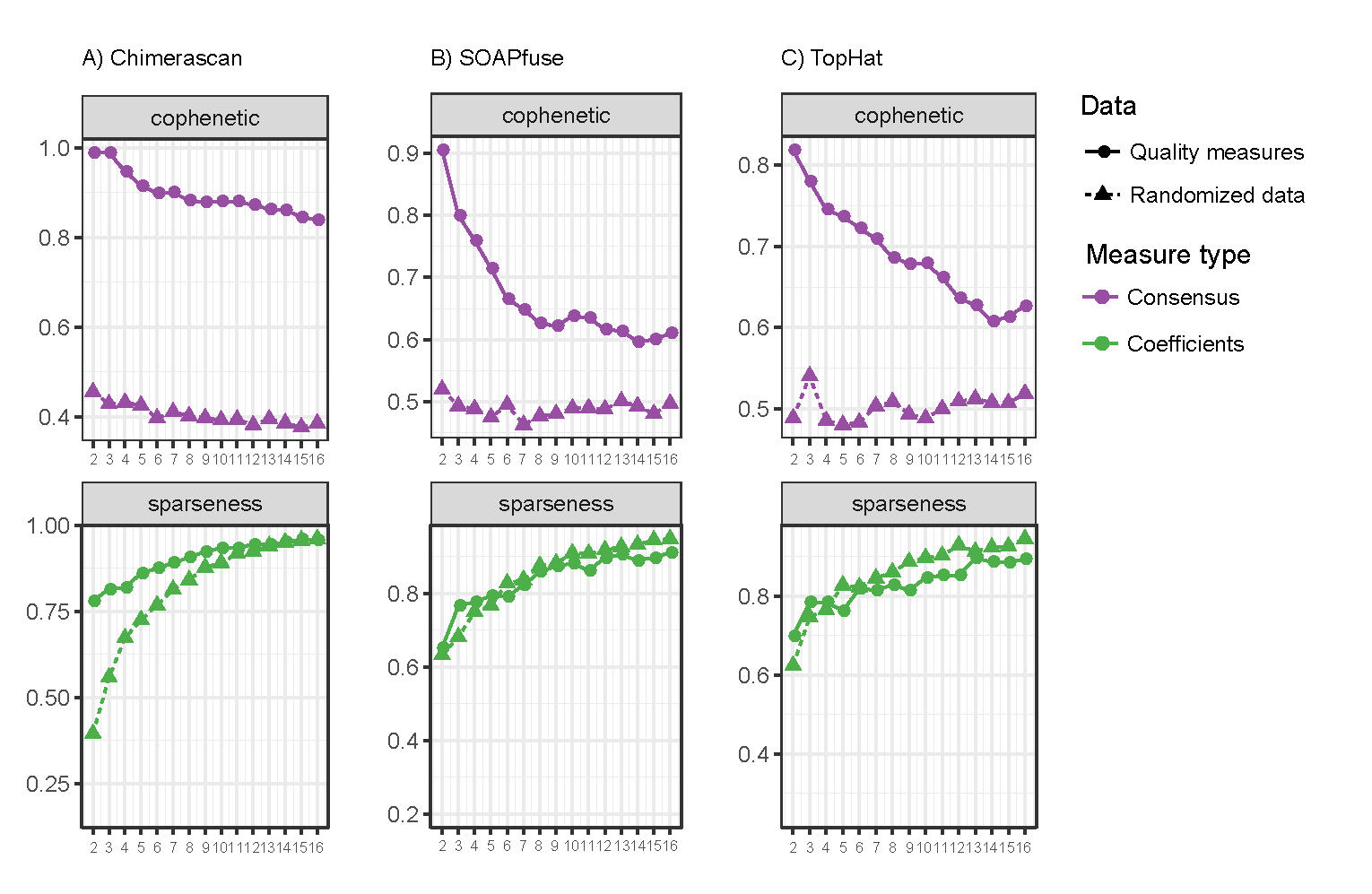

Supplement: Supplementary file 3 [file image3.tif]

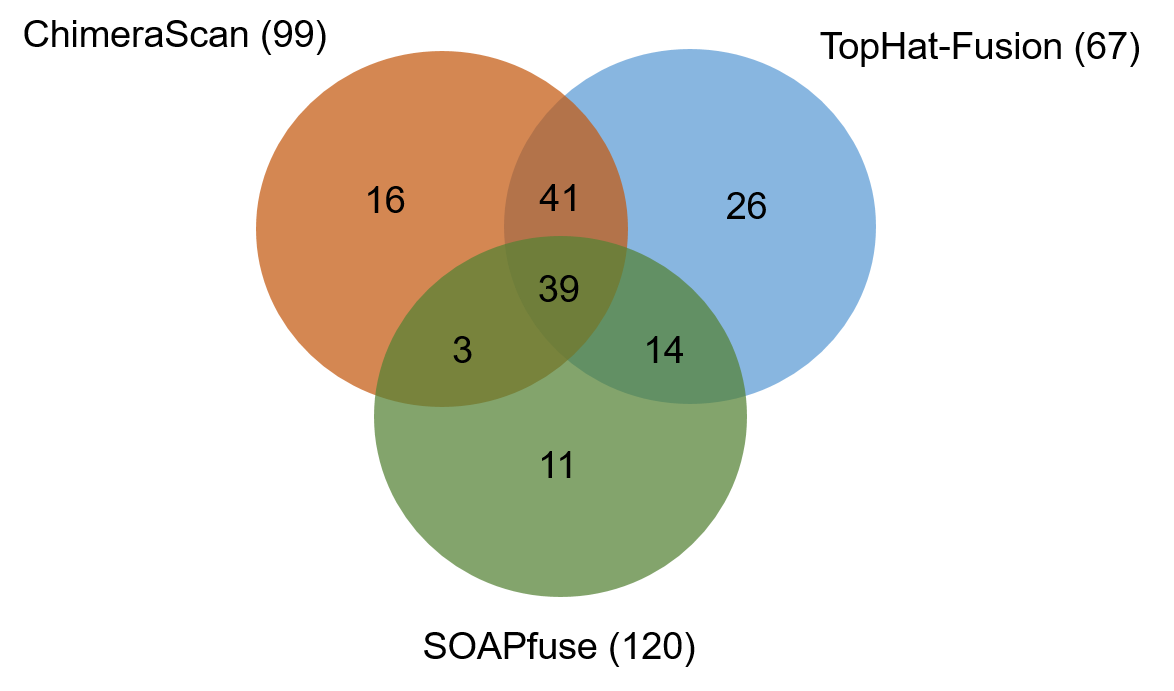

Supplement: Supplementary file 4 [file image4.tif]

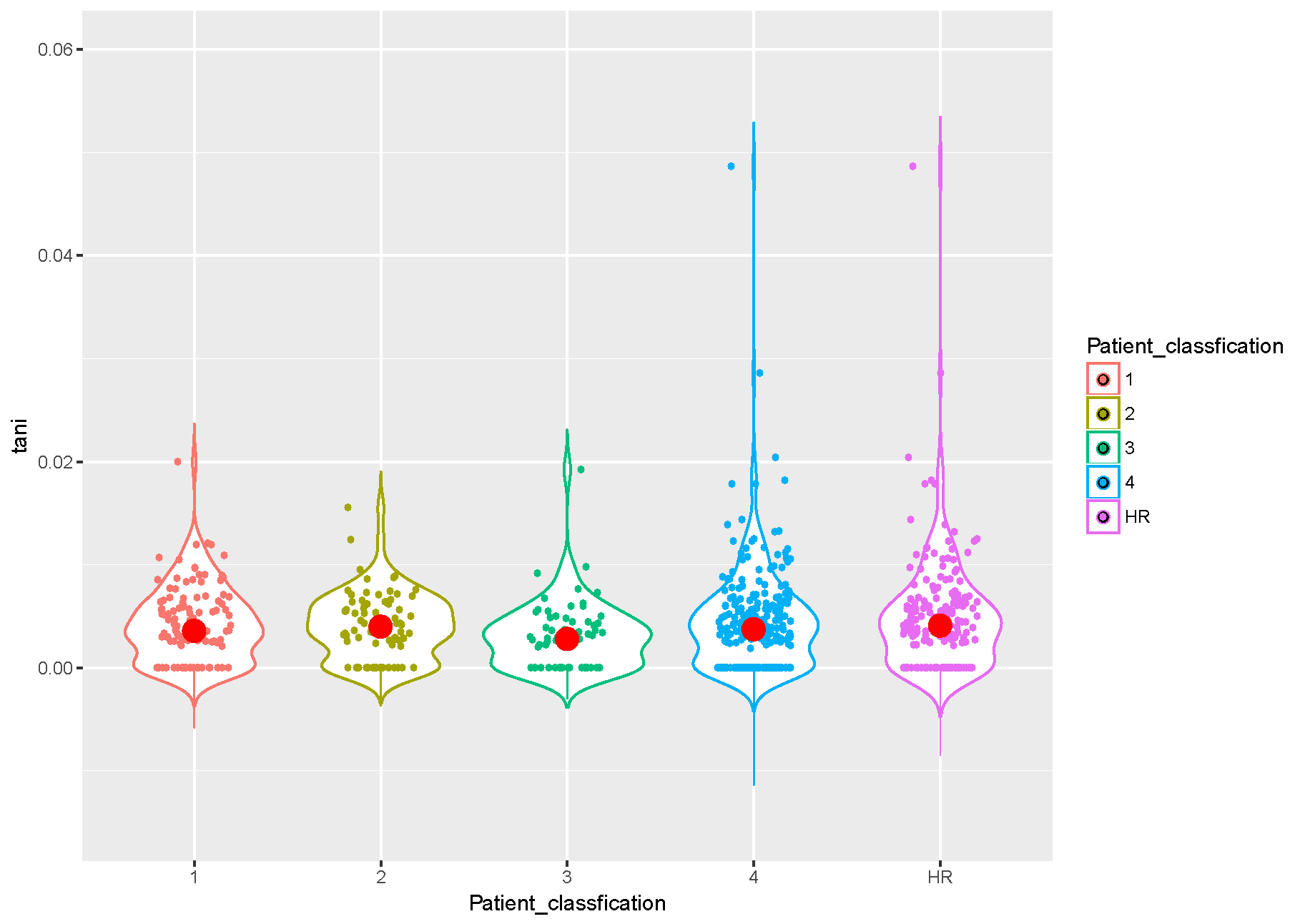

Supplement: Supplementary file 11 [file image2.tiff]
